# Resolving charge-transfer and mass-transfer processes of VO^2+^/VO_2_^+^ redox species across the electrode/electrolyte interface using electrochemical impedance spectroscopy for vanadium redox flow battery†

**DOI:** 10.1039/d0ra05224h

**Published:** 2020-08-20

**Authors:** Pradipkumar Leuaa, Divya Priyadarshani, Debittree Choudhury, Rajan Maurya, Manoj Neergat

**Affiliations:** Department of Energy Science and Engineering, Indian Institute of Technology Bombay (IITB) Powai Mumbai 400076 India nmanoj@iitb.ac.in +91 22 2576 4890 +91 22 2576 7893; Centre for Research in Nanotechnology & Science, Indian Institute of Technology Bombay (IITB) Powai Mumbai 400076 India

## Abstract

Electrochemical impedance spectroscopy is used to investigate the charge-transfer and mass-transfer processes of VO^2+^/VO_2_^+^ (V^4+^/V^5+^) redox species across the carbon-modified glassy carbon disk electrode/electrolyte interface. The features of the EIS patterns depend on the potential, concentrations of the redox species and mass-transport conditions at the electrode/electrolyte interface. With the starting electrolyte containing either only V^4+^ or V^5+^ redox species, EIS shows a straight line capacitor feature, as no oxidation or reduction reaction take place at the measured open circuit potential (OCP). With the electrolyte containing equimolar concentration of V^4+^ and V^5+^, EIS pattern has both charge-transfer and mass-transfer features at the equilibrium potential. The features of the charge-transfer process are observed to be influenced by the mass-transfer process. Optimum concentrations of the V^4+^/V^5+^ redox species and supporting H_2_SO_4_ electrolyte are required to resolve the EIS features corresponding to the underlying physical processes. The semi-infinite linear diffusion characteristics of the V^4+^/V^5+^ redox species observed with a static condition of the electrode converges to that of a finite diffusion under hydrodynamic condition.

## Abstract 2

Effect of redox species concentration across the electrode/electrolyte interface on the EIS features.

## Graphical abstract

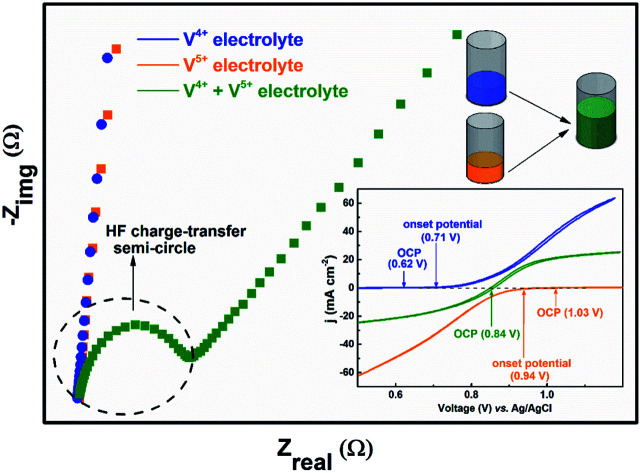

## 1. Introduction

Redox flow batteries (RFB) are one of the most promising large-scale electrochemical energy storage devices due to their excellent energy efficiency, long cycle life, and uncoupled power density and energy density. In particular, the vanadium redox flow battery (VRFB) has apparent advantages over other RFBs, as it employs vanadium electrolyte in both the half-cells, and eliminates the cross-contamination effect through the membrane, resulting in lower electrolyte replacement cost.^1–4^ However, one of the drawbacks of VRFB is its low power density due to the sluggish electron-transfer kinetics of the vanadium redox reactions. Therefore, there is a consistent effort towards improving the electrode kinetics for vanadium redox reactions using either alternative materials with improved properties or by modification of the existing electrode materials.^1–4^ At the same time, a thorough investigation of the electrode kinetics is imperative to establish the effectiveness of the materials towards electron-transfer reactions.

In the literature, the electrochemical impedance spectroscopy (EIS) is extensively used for the comparison of the electrode kinetics.^5–17^ The charge-transfer resistance (*R*_ct_) obtained from the EIS is used to estimate the electrode kinetics (*i.e.*, lower the *R*_ct_ better the kinetics of the redox reactions on the electrode material surface).^5–17^ EIS allows to distinguish various underlying physical processes of different relaxation frequencies,^18–24^ and in the literature, the EIS patterns are reported at the open circuit potential (OCP) or at finite overpotential.^8–17^ At times, EIS is reported with the electrolyte containing only one of the species of the redox couple (either V^4+^ or V^5+^) and not a solution of both.^8–17^ Moreover, prior recording the EIS, electrode is usually subjected to the potential cycling to activate the electrode surface and to achieve maximum wettability.^25,26^ Potential cycling changes the concentration of redox species^27^ and therefore, the EIS recorded just after potential cycling may not give reproducible features. It is a challenging task to derive any conclusion from the EIS recorded at different potentials reported by various authors in the literature. Often, the charge-transfer and mass-transfer processes do not get resolved, if the concentration of the redox species and supporting electrolyte are not optimized; in such cases, only one distorted semi-circle may appear in the EIS pattern and one may interpret it wrongly as charge-transfer process.

Therefore, in this manuscript, evolution of EIS features with the applied potential on a typical carbon material (Vulcan XC-72 carbon-black in RDE configuration) is illustrated using VO^2+^/VO_2_^+^ (V^4+^/V^5+^) redox couple as a redox probe. Voltammograms in three different electrolytes (containing only oxidized species (V^5+^), only reduced species (V^4+^), and with equimolar concentration of both the species (V^4+^ and V^5+^) of the redox couple) are used to elucidate the importance of recording EIS at equilibrium potential (EP). It is shown that the equimolar concentration of both the species of the redox couple in the electrolyte is essential to maintain the equilibrium across the electrode surface. The EIS has to be recorded at EP to obtain reliable and reproducible features and to compare the electrode kinetics. The EIS features depend on concentration of the active species, concentration of the supporting electrolyte and mass-transport conditions. Based on the mass-transport conditions, finite and semi-infinite mass-transport of active species are observed. Also, the role of redox species and supporting electrolyte concentrations in resolving the charge-transfer and mass-transfer processes is underlined. Overall, the precise conditions for the proper comparison of the EIS are derived.

## 2. Experimental details

### 2.1 Materials

Vanadium (V) oxide (V_2_O_5_, 99.6% purity) from Sigma Aldrich; isopropanol (C_3_H_7_OH, 99.5% purity) and sulfuric acid (H_2_SO_4_, 98% GR) from Merck were used as-received without any further purification. High purity (18.2 MΩ) de-ionized (DI) water was obtained from Direct Q Millipore.

### 2.2 Electrolyte preparation

In this work, three different electrolytes (VO^2+^ solution, VO_2_^+^ solution and an equimolar concentration of VO^2+^ and VO_2_^+^) of various concentrations were used for the electrochemical measurements. H_2_SO_4_ solutions of different concentrations (1, 2, 3, 4, and 5 M) were used as the supporting electrolyte in all the three vanadium electrolytes. VO_2_^+^ electrolyte was prepared by dissolving V_2_O_5_ in 3 M H_2_SO_4_ solution. The VO^2+^ electrolyte was prepared by electrochemical reduction of VO_2_^+^ ions. For the simplicity of the text, VO^2+^ and VO_2_^+^ are mentioned as V^4+^ and V^5+^, respectively, throughout the manuscript.

### 2.3 Electrode preparation

The thin-film electrode was prepared by the method reported in the literature.^28^ Carbon-black (5 mg) was dispersed in 5 mL DI water. Subsequently, 10 mL of iso-propanol was added to the mixture and it was ultra-sonicated for 30 min to get a free-flowing smooth ink. A measured volume of the ink was drop-cast using a micro-pipette on a polished glassy-carbon disk electrode (GCE, area 0.196 cm^2^) to get a carbon-black loading of 128 μg cm^−2^. The surface of the working electrode was then air-dried for 2 h prior to the electrochemical measurements.

### 2.4 Electrochemical characterization

The cyclic voltammograms (CVs) were recorded in a conventional three-electrode rotating disk electrode (RDE) configuration using a “WaveDriver 20” bipotentiostat (Pine Research Instruments, USA). The prepared thin-film electrode was the working electrode, Ag/AgCl (saturated KCl) was the reference electrode and a platinum wire was the counter electrode. CV and polarization curves were recorded at 20 mV s^−1^ scan rate. An SP-300 potentiostat (from BioLogic Science Instruments (Seyssinet-Pariset, France)) was used to record the impedance spectra. All the experiments were conducted in a three-electrode thin-film RDE (TF-RDE) configuration with an AC amplitude of 10 mV rms by sweeping the frequency from 20 kHz to 50 mHz at 10 points per decade. The impedance spectra were fitted using complex non-linear least square (CNLS) method with “ZSimpWin” software from Solartron.

When equimolar concentration of both the species of the redox couple is present in the electrolyte, a true equilibrium is established. In such case, the formal equilibrium potential (EP) can be calculated from the thermodynamic data using Nernst equation. On the other hand, when concentration of one of the species in the redox couple is unknown (for *e.g.* after cycling the electrode), or when the supporting electrolyte contains only one of the redox species at the beginning of the experiment, an equilibrium potential is not defined by the Nernst equation. But a potential between the working and reference electrode can be measured using high impedance voltmeter, when no external bias is applied. This potential is called open circuit potential (OCP).^27^ All impedance measurements reported in this manuscript are conducted at either formal EP or OCP, as defined above.

## 3. Results and discussion

### 3.1 CV and EIS in the equimolar (0.2 M) concentration of V^4+^ and V^5+^ in 3 M H_2_SO_4_ electrolyte

**Fig. 1:**
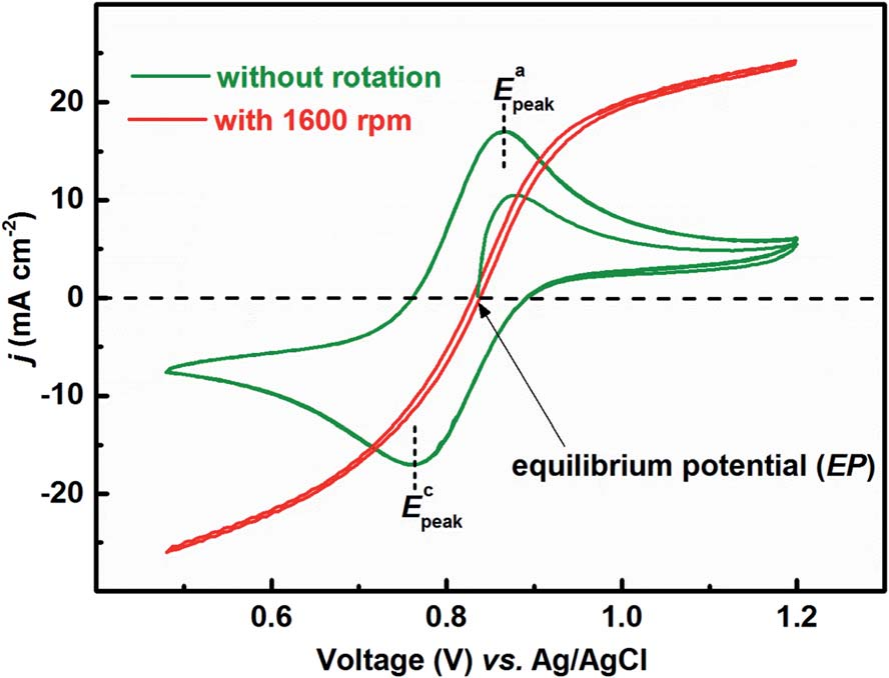
CVs recorded with carbon-modified GCE without rotation and with 1600 rpm at 20 mV s^−1^ scan rate in equimolar (0.2 M) concentration of V^4+^ and V^5+^ in 3 M H_2_SO_4_ electrolyte.

Fig. 1 presents the voltammograms of equimolar (0.2 M) concentration of V^4+^ and V^5+^ in 3 M H_2_SO_4_ electrolyte recorded at 20 mV s^−1^ scan rate. The V^4+^ to V^5+^ oxidation peak is observed at ∼0.88 V and the corresponding reduction peak is at ∼0.78 V; the peak potential difference is ∼0.1 V. The separation of peak potentials (in mV) and peak current ratio are indications of the reversibility of a redox reaction. For a reversible redox reaction, the separation of peak potential should be less than 58/*n* mV and peak current ratio should be 1 at all scan rates, where *n* is the number of electrons involved in the redox reaction.^27^ Therefore, results suggest that VO^2+^/VO_2_^+^ redox couple exhibits a quasi-reversible reaction on carbon-modified GCE. The quasi-reversible reaction indicates that the electron-transfer rate across the electrode/electrolyte interface is slow and higher overpotentials are required to drive the electron-transfer.^27^ The VO^2+^/VO_2_^+^ (V^4+^/V^5+^) is a complex reaction and it involves one electron and two H^+^ transfer in VO_2_^+^ reduction to VO^2+^.

1 VO_2_^+^ + e^−^ + 2H^+^ ↔ VO^2+^ + H_2_O

The EP is chosen as the initial potential for recording the voltammograms. EP is the mid-point potential between the oxidation and the reduction peak potentials.^29^2
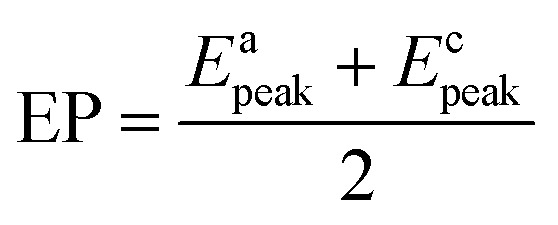
Here, the EP is found to be ∼0.835 V. This is further confirmed by the zero net current at EP with the voltammogram recorded at 1600 rpm (Fig. 1, red curve). The voltammograms recorded without rotation show zero current at the starting point. The concentration of V^4+^ and V^5+^ ions are equal in the bulk of the electrolyte as well as across the electrode surface. But, with the first forward scan, V^4+^ ions oxidize to V^5+^, resulting in an increase of V^5+^ ion concentration (across the electrode surface). Therefore, in the reverse scan, current reaches zero at a higher potential to the initially recorded EP. While, at the end of reverse scan V^5+^ ions gets reduced to V^4+^ and the concentration of V^4+^ ions increases compared to V^5+^. Thus, in the forward scan, current reaches zero at lower potential than the initially recorded EP. Thus, the voltammograms suggest a strong dependence of EP on the relative concentration of the redox species.

**Fig. 2:**
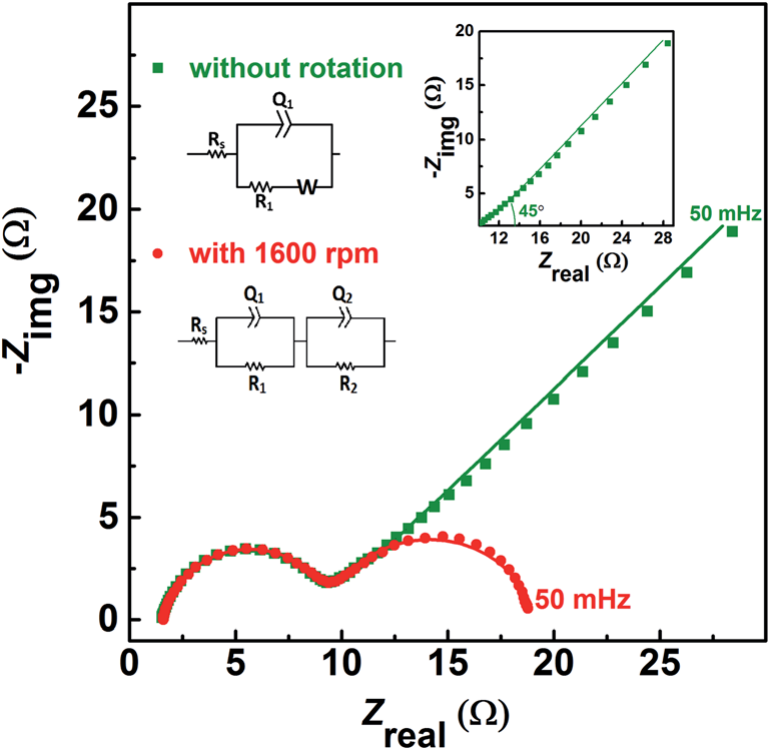
EIS patterns recorded at EP on carbon-modified GCE without rotation and with 1600 rpm in equimolar (0.2 M) concentration of V^4+^ and V^5+^ in 3 M H_2_SO_4_ electrolyte. The symbols and solid lines show the experimental and the fitted data, respectively. The corresponding ECs are shown in the insets. The expanded view of the 45° line is shown in the inset.

Fig. 2 shows the EIS patterns recorded at EP with equimolar (0.2 M) concentration of V^4+^ and V^5+^ in 3 M H_2_SO_4_ electrolyte. The EIS pattern (without rotation of the electrode) shows one high frequency (HF) semi-circle and a low frequency (LF) line corresponding to the charge-transfer process and the semi-infinite linear mass-transport of the vanadium ions, respectively. The semi-infinite linear mass-transport feature is usually fitted with the Warburg element (*W*) and thus, the feature is also known as Warburg diffusion.^29–31^ From the inset to Fig. 2, it is observed that the linear fit to the LF data is at ∼45°. The experimental data points follow the fitted line in the MF range, whereas, a few of the data points appear to be slightly below the linear fit at very low frequencies. The physical reason for the deviation from Warburg behaviour in the LF region is the sluggish diffusion of ions to the porous electrodes. The transport layer impedance depends on the type of the electrode connected to it and for the porous electrodes, as used in this case, the effect of double layer capacitance is not negligible unlike that with planar electrodes.^32,33^ Recently, Juhyun *et al.* reported the effects of nanoparticle geometry and size distribution on diffusion impedance^34^ and Barbero *et al.* showed that the displacement current on the electrodes leads to the transport impedance differing from that of the Warburg's result.^35^ Therefore, the LF data points are slightly deviated from the 45° line as shown in the magnified view of the LF line in the inset to the Fig. 2. At 1600 rpm, the recorded EIS pattern features two semi-circles (see Fig. 2). The HF semi-circle corresponding to the charge-transfer process overlaps in both the spectra. On the other hand, the LF line converges to a semi-circle on recording the EIS under rotation. Therefore, the semi-infinite linear mass-transport feature, in the EIS recorded without rotation converges to a finite mass-transport feature of vanadium ions under the hydrodynamic condition (1600 rpm).

The EIS patterns are fitted with the equivalent circuits (ECs) using complex non-linear least square (CNLS) method. An equivalent circuit consisting of a series combination of *R*_s_ and ((*R*_1_*W*), *Q*_1_) element is used to fit the EIS pattern recorded without rotation, where *R*_s_ is the solution resistance, *R*_1_ is the charge-transfer resistance (*R*_ct_), *Q*_1_ is the constant phase element (CPE) associated with the double layer capacitance (*C*_dl_), and *W* is the Warburg element in series with *R*_1_ (see inset to Fig. 2).^30^ However, a series combination of *R*_s_, HF (*R*_1_, *Q*_1_), and LF (*R*_2_, *Q*_2_) elements is used to fit the EIS pattern recorded under hydrodynamic conditions of the electrode. The HF (*R*_1_, *Q*_1_) element is associated with the electron-transfer process through the electrode–electrolyte interface. The LF (*R*_2_, *Q*_2_) element is associated with the transport of redox species, where *R*_2_ and *Q*_2_ represent the resistance to the transport of redox species (*R*_d_) and CPE associated with the diffuse layer capacitance (*C*_d_), respectively. Also, the quality of the fit is poor with a pure capacitor, and therefore, a constant phase element (CPE) is used to fit the experimental data (see Fig. S1†). The physical reason for replacing capacitor with CPE is the frequency dependence of the measured electrode–electrolyte interfacial capacitance.^36,37^ The CPE behavior is attributed to surface inhomogeneity, reactivity, and current and potential distributions associated with the electrode geometry.^36,37^ Recently, Martin *et al.* attributed the CPE behaviour to the presence of trace amounts of impurities in solutions.^38^

In the literature, sometimes a Warburg short (*W*_s_) element is also used to fit the EIS feature because of finite transport of redox species observed under hydrodynamic conditions of the electrode.^39,40^ Therefore, the EIS pattern is fitted with the series combination of *R*_s_ and the ((*R*_1_*W*_s_), *Q*_1_) element (see Fig. S2a†). The quality of the fit with the *W*_s_ element is poor compared to that of the (*R*_2_, *Q*_2_) element and it is justified with the Fisher–Snedecor test (F-test) (see Fig. S2 and relevant text in the ESI†).^19^ Recently, we reported the transport mechanism of redox species across the porous thin-film electrode/electrolyte interface and explained the conditions in which either *W*_s_ element or (*R*_2_, *Q*_2_) elements should be used to fit the finite transport feature.^30^

Similar EIS features with equimolar concentration of V^4+^/V^5+^ redox species at EP are reported in the literature.^29,41,42^ However, several others have reported such features (one semi-circle and ∼45° line) by recording EIS even at measured OCP or at some finite overpotential in the electrolyte containing one of the species of the redox couple (either V^4+^ or V^5+^) and not a solution of both.^8–17^ The EIS features strongly depend on applied potential and electrolyte concentration. Therefore, it is imperative to understand the EIS recorded at EP, or at a finite potential.

### 3.2 EIS at OCP with 0.2 M V^5+^ electrolyte

**Fig. 3:**
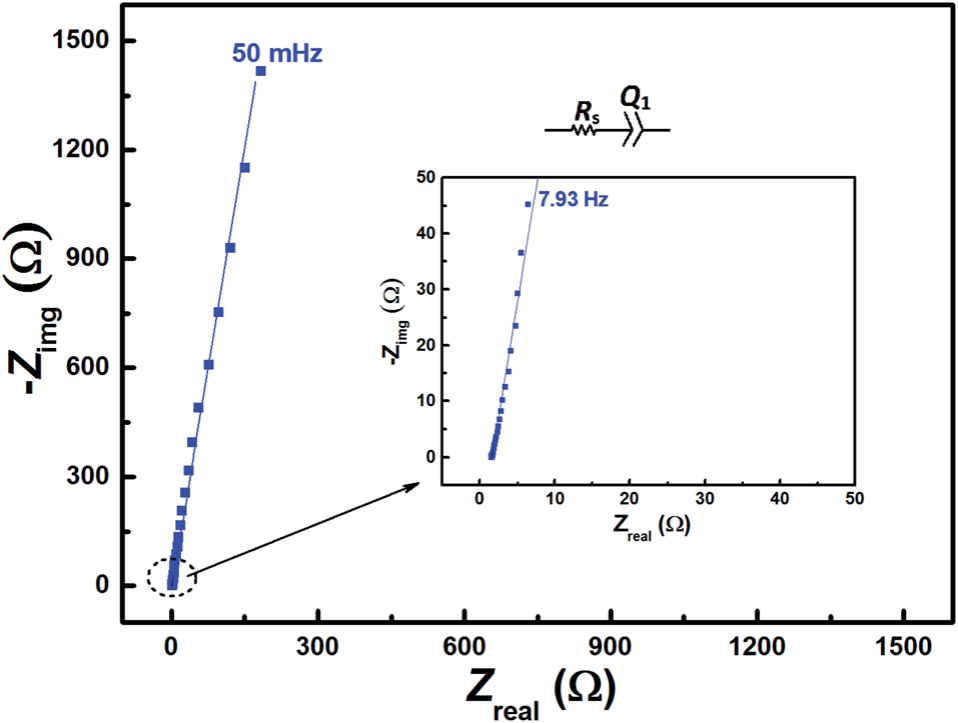
EIS patterns recorded at OCP on carbon-modified GCE without rotation in 0.2 M V^5+^ in 3 M H_2_SO_4_ electrolyte.

The EIS pattern recorded at measured OCP with 0.2 M V^5+^ in 3 M H_2_SO_4_ electrolyte is shown in Fig. 3. In the EIS pattern, only a straight line (slightly tilted away from the *y*-axis) is observed. This indicates a capacitive behaviour of the electrode and charge-transfer reactions such as oxidation and reduction do not take place at OCP. An ideal electrochemical capacitor shows a straight line parallel to the *y*-axis and the non-ideality originates from the surface roughness, reactivity, and porosity of the electrode and its non-uniform current distribution as reported in the literature.^43–45^ Recently, Martin *et al.* attributed the non-ideal behaviour to the presence of trace amounts of impurities in solutions.^38^ Therefore, the observed straight line is slightly tilted away from the *y*-axis.

The carbon electrochemical capacitor usually features equivalent series resistance (ESR) and equivalent distributed resistance (EDR).^23,24^ In the present study, the ESR is negligible (∼1.8 Ω) and the typical HF EDR features are not observed (see the inset to Fig. 3) because of the very high concentration of the acid solution (3 M H_2_SO_4_). The EIS pattern recorded at OCP with V^5+^ electrolyte (Fig. 3) is completely different from that at EP with equimolar concentration of V^4+^ and V^5+^ (Fig. 2). As discussed earlier, in the literature, one semi-circle and semi-infinite linear mass-transport features of vanadium ions are also reported for the EIS recorded at the OCP in the electrolyte containing one of the species of the redox couple (either V^4+^ or V^5+^).^8–12^ Therefore, it is pertinent to understand the OCP, its changes with the concentration of the redox species, and its effect on the EIS features.

### 3.3 Change in the OCP with the concentration of the redox species

**Fig. 4:**
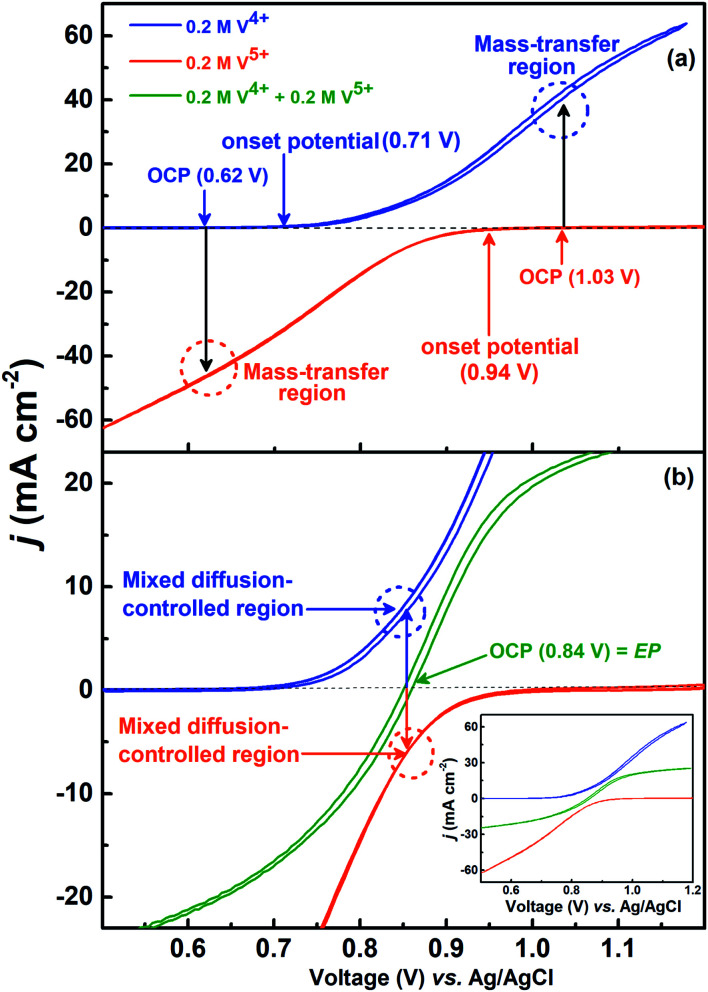
CVs recorded on carbon-modified GCE at 20 mV s^−1^ scan rate with 1600 rpm in 0.2 M V^4+^ and 0.2 M V^5+^ (a) and equimolar (0.2 M) concentration of V^4+^ and V^5+^ electrolytes (magnified view) (b); the complete CVs of V^4+^ and V^5+^ are included in the inset to (b) for comparison.

The voltammograms recorded with 0.2 M V^4+^ and 0.2 M V^5+^ electrolytes at 1600 rpm are shown in Fig. 4(a). The onset potential for the oxidation of V^4+^ is ∼0.71 V (blue curve), and that for the reduction of V^5+^ is ∼0.94 V (orange curve). The OCP measured prior recording the voltammograms was ∼0.62 V for V^4+^ electrolyte and ∼1.03 V for V^5+^ electrolyte. Hence, the OCP is away from the onset potential with both the electrolytes, and it suggests that no oxidation or reduction reactions can take place at OCP. Therefore, the EIS pattern recorded at the OCP with 0.2 M V^5+^ electrolyte shows a straight line capacitive feature and not the charge-transfer semi-circle (see Fig. 3).

For the electrolyte containing equimolar concentration of V^4+^ and V^5+^, the EP is observed to be 0.84 V, (Fig. 4(b)) at the mid-point potential of the oxidation and reduction peak potentials of V^4+^ and V^5+^, respectively (see Fig. 1). Also, the EP is in the mixed diffusion-controlled region as shown in Fig. 4(b). Therefore, the EIS pattern recorded at EP in the electrolyte containing equimolar concentration of V^4+^ and V^5+^ shows features of charge-transfer (semi-circle) and semi-infinite linear mass-transport (45° line) of vanadium ions (see Fig. 2). Here, the oxidation and reduction currents increase with the overpotential and do not appear to reach a steady state for the 0.2 M V^4+^ (blue trace) and 0.2 M V^5+^ (orange trace) electrolytes, as compared to the oxidation and reduction currents of the equimolar (0.2 M) solution of V^4+^ and V^5+^ (green trace in inset to Fig. 4(b)). Also, the oxidation current in the 0.2 M V^4+^ electrolyte and the reduction current in the 0.2 M V^5+^ electrolyte are higher than the oxidation and reduction currents observed for the equimolar (0.2 M) solution of V^4+^ and V^5+^ electrolyte. This can be due to the complex nature of the VO^2+^/VO_2_^+^ redox reaction.^46–48^

**Fig. 5:**
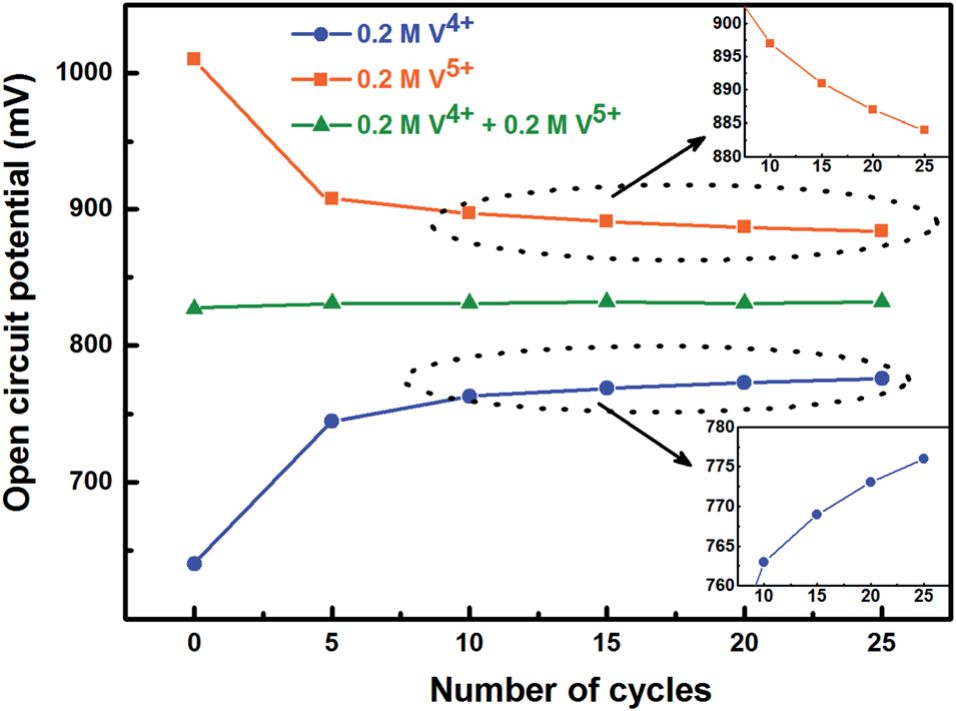
Change in measured OCP after recording CV without rotation on carbon-modified GCE at 20 mV s^−1^ scan rate for regular number of cycles.

To activate the electrode surface, potential cycling is often carried out prior recording the EIS patterns. Thus, the concentration of the redox species changes across the electrode surface and it affects the measured OCP. To study the deviation in OCP, the concentrations of V^4+^ and V^5+^ ions were varied by potential cycling without rotation (from 0.48 V to 1.2 V) and its effect on OCP was recorded and shown in Fig. 5. The final ratio of the V^4+^ and V^5+^ across the electrode surface is largely determined by the potential at which the CV scanning is terminated. Therefore, all the CVs are recorded such that it starts at EP (0.84 V) and terminates at the same potential as shown in Fig. 1.

With the 0.2 M V^5+^ electrolyte, the OCP decreases with the potential cycling due to the V^5+^ reduction to V^4+^ across the electrode surface; the V^4+^/V^5+^ redox couple exhibits a quasi-reversible reaction (see Fig. 1). Both concentration of V^4+^ ions across the electrode surface and the OCP stabilize after certain number of cycles (∼25) (see Fig. S3†). Similarly, with the V^4+^ electrolyte, OCP increases with potential cycling due to the increase in V^5+^ ion concentration across the electrode surface. With the electrolyte containing equimolar concentration of V^4+^ and V^5+^, the EP is found to be stable with the potential cycling. Here, the concentration of V^4+^ and V^5+^ is equal in the bulk of the electrolyte as well as across the electrode surface. Thus, after potential cycling, concentration would not change to affect the EP. With the potential cycling, the OCP recorded in 0.2 M V^4+^ (blue trace) and 0.2 M V^5+^ (orange trace) electrolytes approaches towards the EP (green trace) recorded in equimolar (0.2 M) solution of V^4+^ and V^5+^ electrolytes. However, OCP in V^4+^ and V^5+^ electrolytes never reaches to the EP. In the V^5+^ electrolyte, the V^5+^ ions reduces to V^4+^ with the potential cycling, and the overall concentration of V^5+^ and V^4+^ across the electrode surface shifts the EP to a new value, which is lower than the initial value as discussed in Section 3.1 (see Fig. 1 and the relevant text). With the time, V^4+^ ions diffuses into the bulk of the electrolyte and thus the overall concentration of V^5+^ ions across the electrode surface remains higher than that of V^4+^ ions and the measured OCP (orange trace) does not reach to EP (green trace). Similar arguments holds for the V^4+^ electrolyte as well.

The concentration of V^4+^ and V^5+^ can be simply varied by holding the working electrode at any potential between the OCPs of the V^4+^ and V^5+^ electrolytes. Therefore, the working electrode was held at EP (0.84 V) which is a large overpotential for the V^4+^ oxidation as well as V^5+^ reduction (see Fig. 4) and the change in OCP was recorded with time. The changes in the value of OCP with the potential holding in 0.2 M V^4+^, 0.2 M V^5+^ and equimolar (0.2 M) solution of V^4+^ and V^5+^ are similar to the results observed with the potential cycling and it is shown in Fig. S4.†

### 3.4 EIS at OCP after potential cycling in 0.2 M V^5+^ electrolyte

**Fig. 6:**
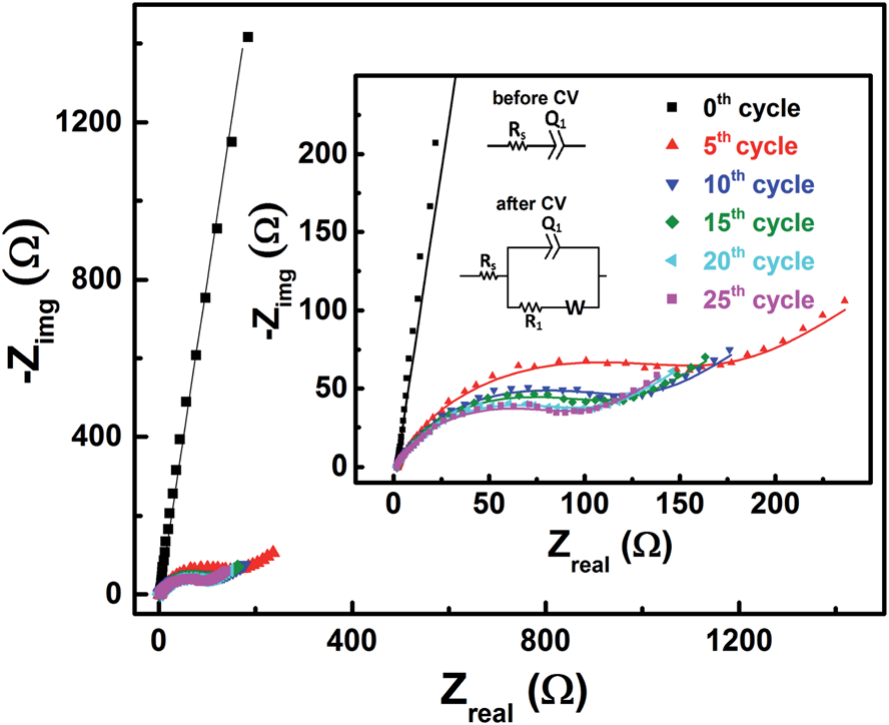
EIS patterns recorded at OCP on carbon-modified GCE without rotation in 0.2 M V^5+^ electrolyte after every 5 cycles. The symbols and solid lines show the experimental and the fitted data, respectively. The corresponding EC is shown in the inset.

EIS patterns recorded at OCP with 0.2 M V^5+^ electrolyte after every 5 cycles are shown in Fig. 6. Prior to cycling, as already discussed in Section 3.2 (see Fig. 3), the recorded EIS pattern shows a straight line feature. After five cycles, the OCP shifts to lower potential (see Fig. 5) and the features of the EIS pattern recorded are different with a semi-circle and a 45° straight line. Here, it is to be noted that the semi-circle is not due to the V^5+^ reduction to V^4+^, but it is primarily a result of V^4+^ ions (generated across the electrode surface during CV) oxidation to V^5+^ since the OCP is a high overpotential for the oxidation of V^4+^ (see Fig. 4(a)). With further cycling, V^4+^ ion concentration increases across the electrode surface and the semi-circle diameter is observed to decrease. After 25 cycles, concentration of V^4+^ ions stabilizes across the electrode surface, and therefore, no further changes in the measured OCP and EIS features are observed. Similar features are observed for the EIS recorded at measured OCP after holding the working electrode at EP in 0.2 M V^5+^ electrolyte (see Fig. S5 in the SI†). This confirms that the EIS features strongly depends on the concentration of the redox species across the electrode surface.

As shown in the Fig. 4, the OCP with V^5+^ electrolyte is a high overpotential for the oxidation of V^4+^ ions present across the electrode surface and it is in the mass-transfer region of the polarization curve recorded with V^4+^ electrolyte. Therefore, recording the EIS patterns at OCP in an electrolyte containing only a single redox species (either V^4+^ or V^5+^) of the redox couple may lead to wrong information. Depending on the surface area and pore volume of the electrode material, concentration of ions across the electrode surface would be different even after recording the CVs for equal number of cycles. Furthermore, the ions across the electrode surface disperse to the bulk of the solution with time. Hence, the features of the EIS patterns just after recording the CV and after certain time would be different. Therefore, the concentration of both the species (V^4+^ and V^5+^) of the redox couple should be equal in the electrolyte to maintain the equilibrium across the electrode surface.

The above experiments were carried out with the equimolar (0.2 M) concentration of V^4+^ and V^5+^ in 3 M H_2_SO_4_ electrolyte, which was optimized by recording CVs and EIS. The effect of the vanadium ions and H_2_SO_4_ concentration on CV and EIS is discussed in the following sections (Sections 3.5 and 3.6).

### 3.5 Effect of redox species concentration

**Fig. 7:**
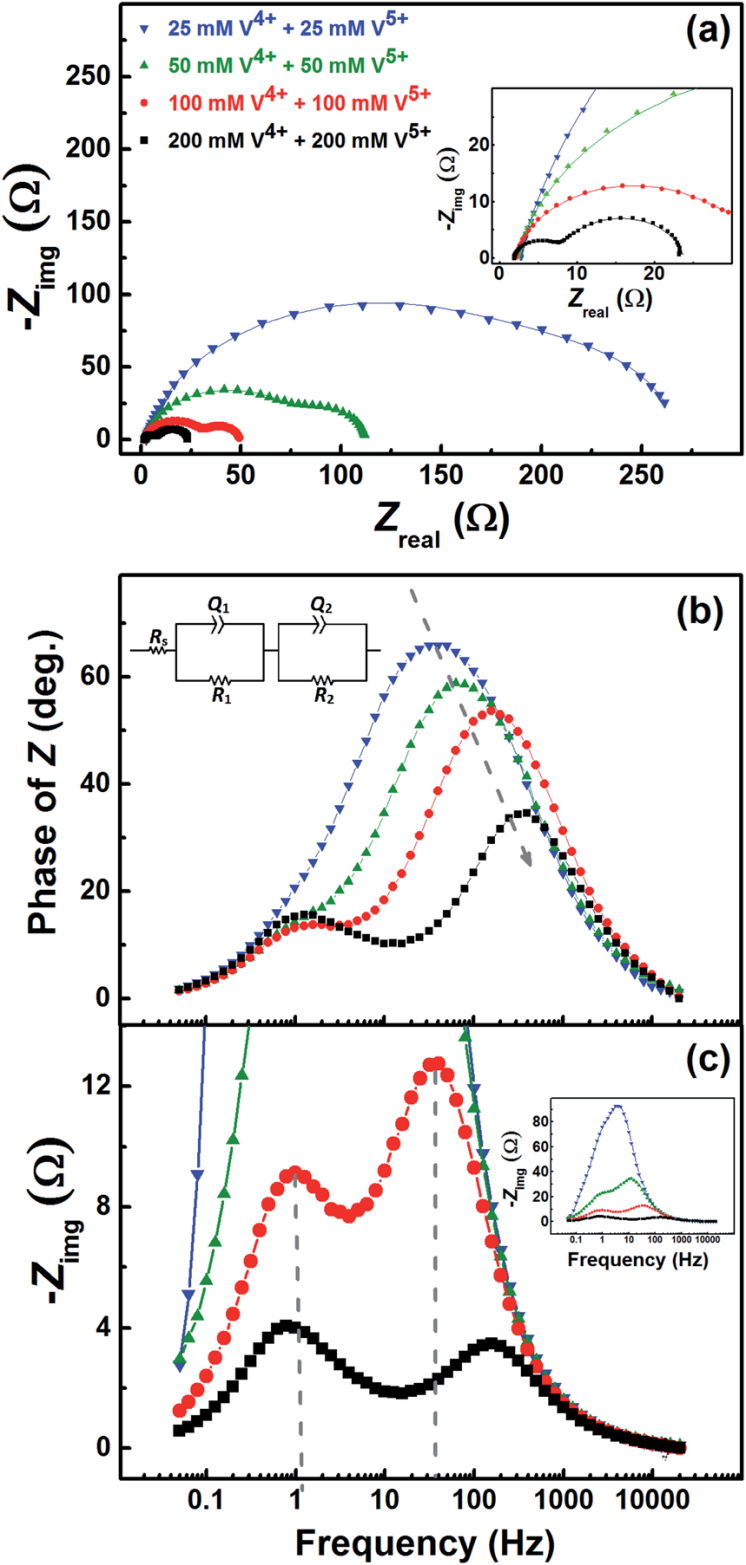
(a) EIS patterns recorded at EP on carbon-modified GCE with 1600 rpm in the equimolar concentration of V^4+^ and V^5+^ in 3 M H_2_SO_4_ electrolytes. The corresponding Bode phase plots and Bode imaginary plots are shown in (b) and (c), respectively, along with the equivalent circuit (inset). The symbols and solid lines show the experimental and the fitted data, respectively.

Fig. 7 shows the EIS patterns recorded at the EP with equimolar concentration of V^4+^ and V^5+^ in 3 M H_2_SO_4_. Two well-resolved semi-circles appear at high concentration (200 mM) of the vanadium ions (V^4+^/V^5+^) and they merge at lower concentrations. The EIS patterns are fitted with an EC consisting of a solution resistance *R*_s_ in series with two (*R*, *Q*) elements; the obtained values of charge-transfer resistance (*R*_ct_) and mass-transfer resistance (*R*_d_) are shown in Table S3.† It is observed that the semi-circle diameter increases with the decrease in vanadium ions concentration, and it indicates rise in charge-transfer and mass-transfer resistance (see Table S3†). Further, in the Bode phase (Fig. 7(b)) plots, single peak corresponding to charge-transfer process is observed at the MF region for the lower concentration and the peak position shifts to higher frequency with the rise in V^4+^/V^5+^ concentration. It suggest that the charge-transfer becomes a limiting process at the lower concentration of V^4+^/V^5+^ and thus it is pushed to lower frequency. This can be the reason for the poor resolution of semi-circles in the Nyquist plots at lower concentrations. For the higher concentration of V^4+^/V^5+^, two well-separated peaks (HF and LF) corresponding to the charge-transfer and mass-transfer processes are observed.

The corresponding Bode imaginary plots are shown in Fig. 7(c). Only a single peak is observed in the MF region for the low concentration (25 mM) of V^4+^/V^5+^, similar to the Bode phase plots. At higher concentrations (50 mM and above), it splits into two peaks; with increase in the V^4+^/V^5+^ concentration, the HF peak shifts to further higher frequencies and LF peak to further low frequencies. Thus, two well-resolved semi-circles are observed in the Nyquist plots with the higher concentration of V^4+^/V^5+^.

### 3.6 The role of supporting electrolyte

The redox reactions are usually investigated in acidic medium due to the low solubility of the salts in water. Ions from the supporting electrolyte also may take part in the charge-transfer reaction. In such cases, it is desirable to optimise the supporting electrolyte concentration; otherwise, it may limit the reaction. In this study, H_2_SO_4_ is used as a supporting electrolyte. So far, all the experiments discussed above were carried out in optimized H_2_SO_4_ concentration (3 M).

**Fig. 8:**
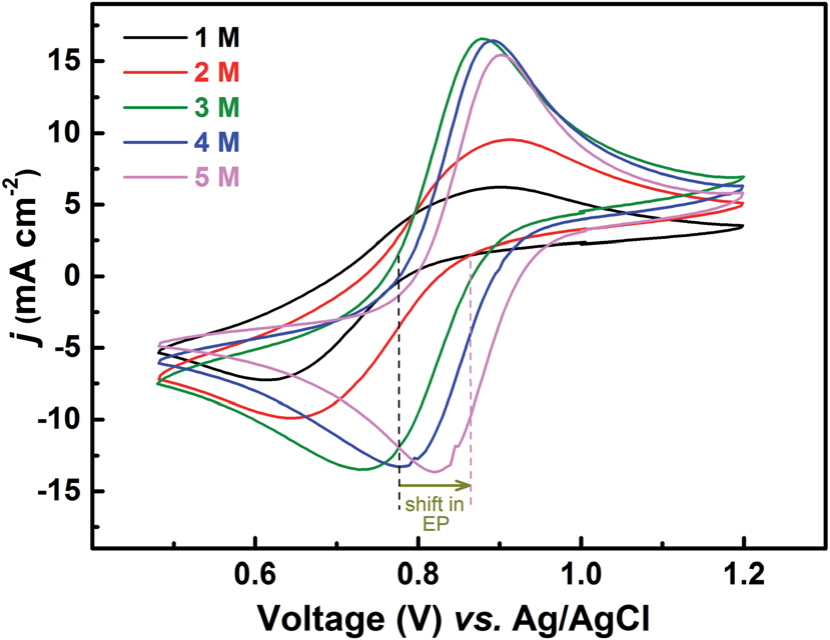
CVs recorded on carbon-modified GCE at 20 mV s^−1^ scan rate in equimolar (0.2 M) solution of V^4+^ and V^5+^ in H_2_SO_4_ electrolyte of different concentrations. The dotted lines show the shift in EP with supporting electrolyte (H_2_SO_4_) concentration.

**Fig. 9:**
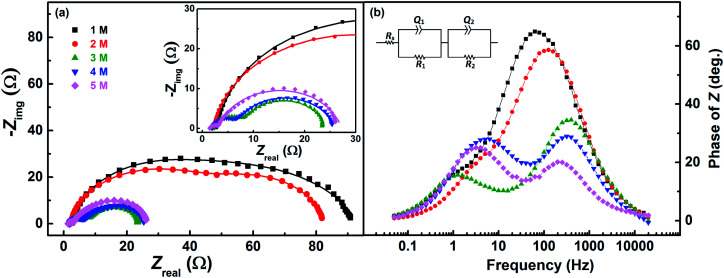
(a) EIS recorded at EP on carbon-modified GCE with 1600 rpm in equimolar (0.2 M) concentration V^4+^ and V^5+^ in H_2_SO_4_ electrolyte of different concentrations. The corresponding Bode phase plots are shown in (b), along with the equivalent circuit (inset). The symbols and solid lines show the experimental and the fitted data, respectively.

Voltammograms recorded with equimolar (0.2 M) solution of V^4+^ and V^5+^ in H_2_SO_4_ electrolyte of different concentrations are shown in Fig. 8. As two H^+^ ions are involved in the V^5+^ (VO_2_^+^) reduction to V^4+^ (VO^2+^), the higher concentration of H_2_SO_4_ (than that of V^5+^), boosts peak current, reduces the peak to peak separation and facilitates the reaction. However, at concentration above 3 M, the reaction slows down perhaps due to rise in viscosity of the electrolyte. From eqn (1), the EP is dependent on the solution pH and it is found to shift with the increase in H_2_SO_4_ concentration. With increase in the SO_4_^2−^ ion concentration in the electrolyte, the specific adsorption of the SO_4_^2−^ changes the potential at the outer Helmholtz plane and increases the rate constant by the well-known Frumkin effect.^49^ The EIS patterns recorded at corresponding EP are shown in Fig. 9. The charge-transfer resistances decrease with H_2_SO_4_ concentration, whereas, the mass-transfer resistance decrease with H_2_SO_4_ concentration up to 3 M, and increases at further higher concentration (4 M and 5 M) due to rise in viscosity of the electrolyte.^50–52^ Similar observations were also reported by Yuehua *et al.*^53^ The solution resistance (*R*_s_) is observed to be decreased with the rise in H_2_SO_4_ concentration. The solution resistance, charge-transfer resistance and mass-transfer resistance obtained from EC fitting are shown in Table S4.†

The semi-circles for the charge-transfer and mass-transfer processes are best resolved at 3 M H_2_SO_4_ concentration which is further supported by Bode phase plots shown in Fig. 9(b). The HF and LF peaks get well-separated on increasing the H_2_SO_4_ concentration. However, due to the rise in viscosity of electrolyte, at concentration above the 3 M H_2_SO_4_, the transport of the ions towards the electrode surface gets hindered and the mass-transfer becomes the limiting process. Therefore, the LF peak is found to be shifted to higher frequencies with the 4 M and 5 M H_2_SO_4_ concentrations, causing poor resolution of charge-transfer and mass-transfer processes. Therefore, it is essential to use the optimized concentration of supporting electrolyte, which is 3 M H_2_SO_4_ in the present case.

## 4. Conclusions

The evolution of EIS patterns with the applied potential, concentration of the redox species, and the concentration of the supporting electrolyte is investigated using the V^4+^/V^5+^ (VO^2+^/VO_2_^+^) redox couple on the carbon-modified GCE. For the electrolyte containing equimolar concentration of V^4+^ and V^5+^, the EIS pattern is characterized by the features of charge-transfer and mass-transfer processes at EP. When concentration of one of the species of the redox couple is not known or when the supporting electrolyte contains only one of the species before the start of the experiment, an OCP is not defined. In such cases, the EIS features will change with potential cycling. Therefore, it is imperative to record EIS at the EP to obtain reproducible EIS pattern and the kinetic parameters. Moreover, the charge-transfer process is observed to be influenced by the mass-transport conditions at the electrode/electrolyte interface. Optimum concentration of V^4+^/V^5+^ and supporting H_2_SO_4_ solution are required to resolve the charge-transfer and mass-transfer processes. At lower concentrations of the V^4+^/V^5+^, charge-transfer becomes a limiting process and shifts towards LF, which results in poor resolution of EIS features. At higher concentrations, charge-transfer process shifts towards higher frequency, resolving it from the LF mass-transfer process. In addition, it is essential to choose optimum concentration of the supporting electrolyte when the ions from the supporting electrolyte solution take part in the charge-transfer process.

## Conflicts of interest

There are no conflicts of interest to declare.

## Supplementary Material

RA-010-D0RA05224H-s001
