# Supplementary material for: Resolving charge-transfer and mass-transfer processes of VO2+/VO2+ redox species across the electrode/electrolyte interface using electrochemical impedance spectroscopy for vanadium redox flow battery
Source: RSC Adv. 2020 Aug 20;10(51):30887–95. doi: 10.1039/d0ra05224h (PMC9056327; doi:10.1039/d0ra05224h)
Supplement: RA-010-D0RA05224H-s001 [file RA-010-D0RA05224H-s001.pdf]

Electronic Supplementary Material (ESI) for RSC Advances.
This journal is © The Royal Society of Chemistry 2020

Resolving Charge-transfer and Mass-transfer Processes of VO^{2+}/VO_2^+ Redox Species Across the Electrode/Electrolyte Interface Using Electrochemical Impedance Spectroscopy for Vanadium Redox Flow Battery

Pradipkumar Leuaa[1], Divya Priyadarshani[2], Debittree Choudhury[1], Rajan Maurya[1], Manoj Neergat[*,1]

[1]Department of Energy Science and Engineering and [2]Centre for Research in Nanotechnology & Science, Indian Institute of Technology Bombay (IITB), Powai, Mumbai 400076, India

Supporting Information (SI)

1. Role of constant phase element (CPE)

Figure S1 shows the EIS of equimolar solution of V^{4+} and V^{5+} on carbon-modified GCE in 3 M H_2SO_4 electrolyte recorded at EP. The fit of the impedance spectrum with pure capacitor is poor (Figure S1(a)). On the other hand, the fit is perfect with a CPE element (Figure S1(b)), instead of capacitor. To account for the distributed behavior of the porous electrode, we have fitted all the data in this manuscript with a constant phase element (CPE).

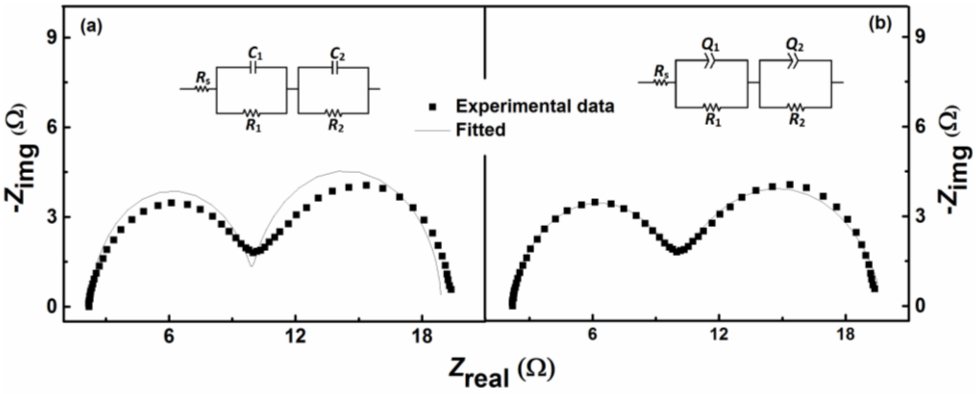

Figure S1. EIS of equimolar concentration of V^{4+} and V^{5+} in 3 M H_2SO_4 electrolyte (a) fitted with pure capacitor and (b) fitted with constant phase element (CPE), recorded at EP on carbon-modified GCE at 1600 rpm. Inset to the figures shows the corresponding equivalent circuit.

2. F-test for the EC of EIS patterns recorded with 1600 rpm

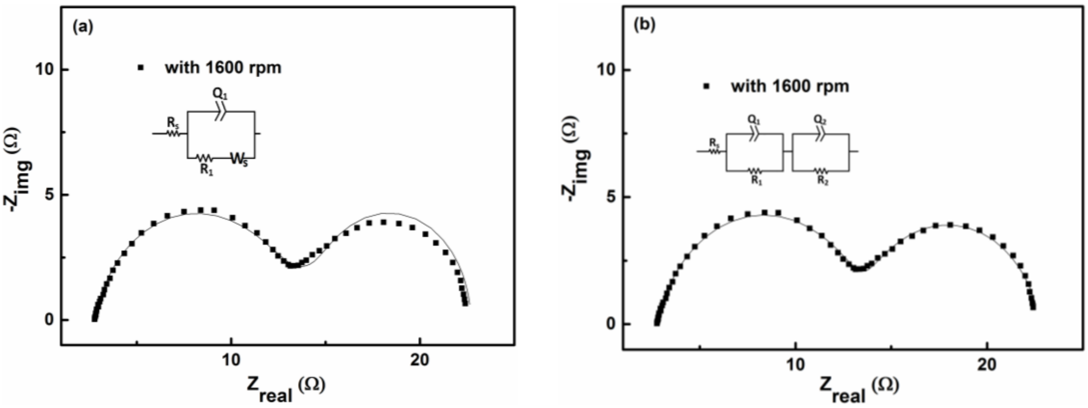

Figure S2. EIS pattern recorded at EP on carbon-modified GCE at 1600 rpm in equimolar (0.2 M) concentration of VO^{2+} and VO_2^+ in 3 M H_2SO_4 electrolyte. The EC used to fit the data is shown in the insets.

Table S1. Parameters obtained by fitting the EIS pattern recorded at EP on carbon-modified GCE at 1600 rpm in equimolar (0.2 M) concentration of VO^{2+} and VO_2^+ in 3 M H_2SO_4 electrolyte. The EC used for fitting the data is shown in Figure S2(a).

Parameter	Obtained fitting value	rel. std. error (%)	x^2 (Chi-square)	Relative error of the measured impedance
R_s	2.803	0.5486	0.0002854	1.69
R_{ct}	10.23	0.9321		
Q_1 (S-sec^ϕ)	0.000283	4.956		
Φ_1	0.8572	0.794		
W_s-Y_o	0.07863	2.165		
W_s-B	0.7342	1.859		

Table S2. Parameters obtained by fitting the EIS pattern recorded at EP on carbon-modified GCE at 1600 rpm in equimolar (0.2 M) concentration of VO^{2+} and VO_2^+ in 3 M H_2SO_4 electrolyte. The EC used for fitting the data is shown in Figure S2(b).

Parameter	Obtained fitting value	rel. std. error (%)	x^2 (Chi-square)	Relative error of the measured impedance
R_s	1.789	0.4131	0.0001562	1.25
R_{ct}	10.78	0.8809		
Q_1 (S-sec^ϕ)	0.0003297	3.688		
Φ_1	0.8407	0.5785		
R_d	9.2	1.97		
Q_2 (S-sec^ϕ)	0.00629	3.071		
Φ_2	0.8725	1.95		

The EIS patterns recorded at 1600 rpm of the electrode are fitted with a series combination of R_s and $(Q(R_1W_s))$ element, where W_s is a finite length transmisive impedance (Warburg short element) placed in series with R_1 (see inset to Figure S2). However, when the LF semi-circle is fitted with (R_2, Q_2) element, then the x^2 (Chi-square) is lower. The F-test also justifies the fitting of LF semi-circle with (R_2, Q_2) element.

Number of degrees of freedom v= 2N-m,

Where N is the number of frequencies, there are 2N measured impedance values (N real and N imaginary), and m is the number of adjustable parameters used in the model.

N=57 points,

k is the number of parameters added in new model.

F(α, k, 2N-m-k) = 3.929844 (calculated in Excel using the function F.INV.RT)

Where, k=1, m=6 and N=57, α = 0.05

$$F_{exp} = \frac{\frac{s_1 - s_2}{(2N-m)-(2N-m-k)}}{\frac{s_2}{2N-m-k}} = \frac{\frac{s_1 - s_2}{k}}{\frac{s_2}{2N-m-k}}$$

$S_1 = 1.69$, $S_2 = 1.25$

$$F_{exp} = \frac{\frac{1.69 - 1.25}{1}}{\frac{1.25}{107}} = 37.66$$

$F_{exp} > F(\alpha, k, 2N\text{-}m\text{-}k)$

Therefore, the model used in the manuscript (fitting the LF semi-circle with (R_2, Q_2) element) can be accepted.

3. Voltammograms of V^{4+} and V^{5+} containing electrolytes

The voltammograms recorded with 0.2 M V^{4+} and 0.2 M V^{5+} electrolytes are shown in **Figure S3**. The voltammograms are reproducible after 20 cycles. The change in the peak currents during the initial 25 cycles of voltammograms is shown as insets to Figure S3(a) and (b).

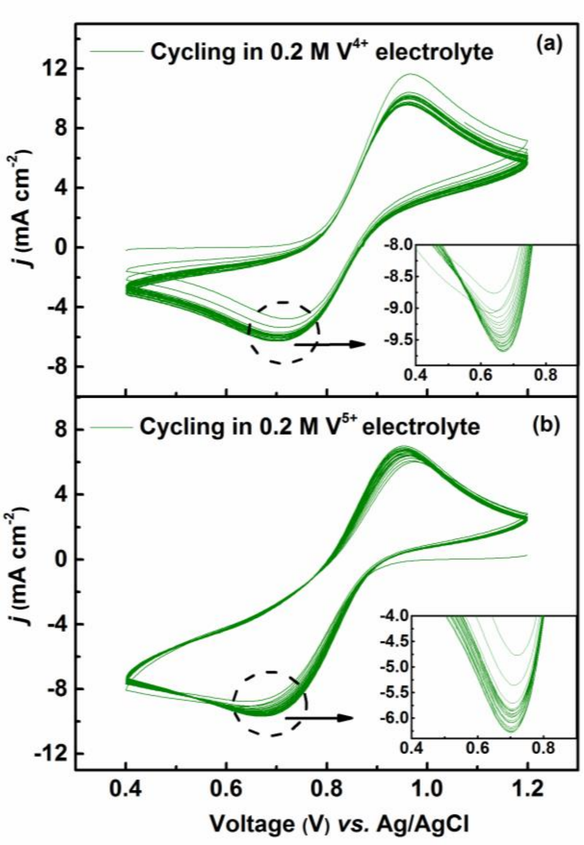

Figure S3. Voltammograms of 0.2 M V^{4+} (a) and 0.2 M V^{5+} (b) in 3 M H_2SO_4 electrolyte recorded at 20 mV s^{-1} scan rate.

4. Change in the OCP with the concentration of the redox species

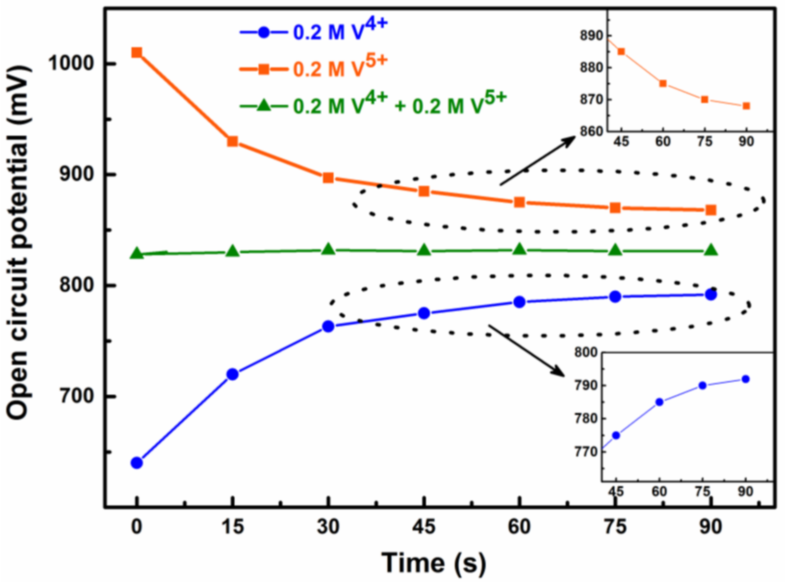

Figure S4. Change in measured OCP with the potential holding of working electrode at 0.84 V without rotation in 0.2 M V^{4+}, 0.2 M V^{5+} and equimolar (0.2 M) solution of V^{4+} and V^{5+} electrolytes.

5. EIS at OCP after potential holding in 0.2 M V^{5+} electrolyte

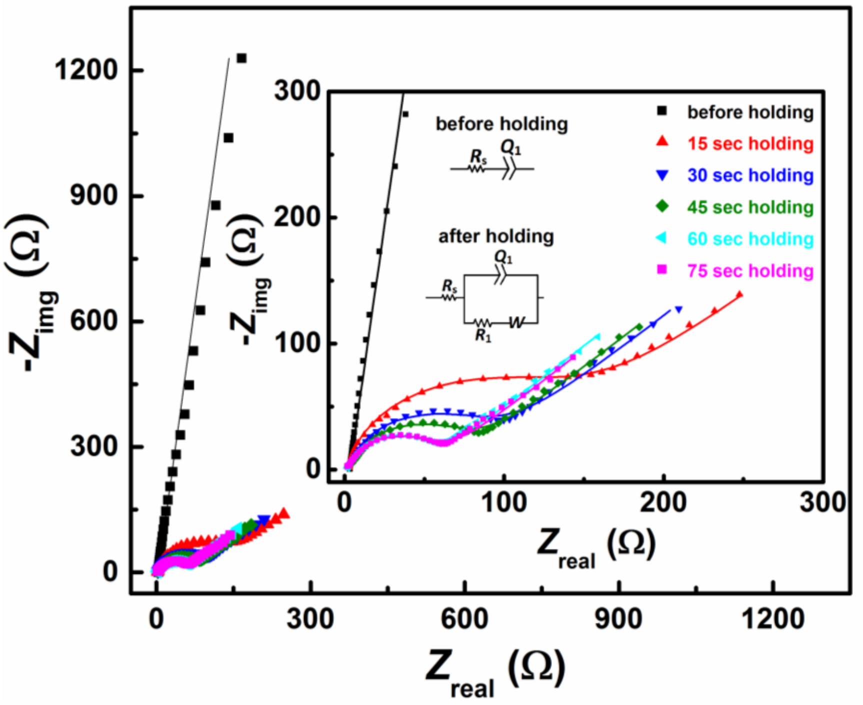

Figure S5. EIS patterns recorded at measured OCP on carbon-modified GCE without rotation in 0.2 M V^{5+} electrolyte after holding the electrode at EP. The symbols and solid

lines show the experimental and the fitted data, respectively. The corresponding EC is shown in the inset.

6. Effect of redox species concentration

Table S3: Charge-transfer and mass-transfer resistance obtained by fitting the EIS patterns of equimolar solution of V^{4+} and V^{5+} in 3 M H_2SO_4 electrolyte.

**Concentration of V^{4+} and V^{5+} (mM) (1:1 ratio)**	**R_{ct} (Ω)**	**C_{dl} (μF)**	**R_2(Ω)**	**C_2 (μF)**
25	203	485	62	3252
50	77	415	33	3917
100	29	348	19	4828
200	7.8	129	13	6613

7. Effect of H_2SO_4 concentration

Table S4: Charge-transfer and mass-transfer resistance obtained by fitting the EIS patterns of equimolar (0.2 M) solution of V^{4+} and V^{5+} with different concentrations of H_2SO_4 electrolyte.

**Concentration of H_2SO_4 (M)**	**R_s (Ω)**	**R_{ct} (Ω)**	**C_{dl} (μF)**	**R_2(Ω)**	**C_2 (μF)**
1	2.83	60	630	31	189
2	2.47	44	137	35	1985
3	2.12	7.8	129	13	6613
4	1.87	4.7	176	19	7710
5	1.45	2.1	254	23	9394
